# Supplementary material for: Factors influencing vaccination of dogs against rabies in South Mugirango Sub-County-Kisii County; Kenya
Source: PLOS Glob Public Health. 2025 May 9;5(5):e0004194. doi: 10.1371/journal.pgph.0004194 (PMC12063882; doi:10.1371/journal.pgph.0004194)
Supplement: Attachment — Submitted filename: Manuscript_Review.docx [file pgph.0004194.s001.docx]

The manuscript, "Factors Influencing Vaccination of Dogs against Rabies within Household Heads in South Mugirango Sub-County-Kisii County; Kenya," investigates the awareness and vaccination practices related to rabies among household heads in South Mugirango, Kenya. The study employs a mixed-methods approach, combining a descriptive cross-sectional survey with 422 household heads and key informant interviews with 22 health officials. Key findings indicate a high level of rabies awareness (94.8%) among respondents, with significant socio-demographic factors influencing vaccination adherence, including age, education level, occupation, and income. However, despite the high awareness, vaccination rates were low, with only 15.9% of respondents reporting recent vaccinations of their dogs. The study highlights a discrepancy between awareness and practice, attributing low vaccination rates to logistical challenges, inadequate funding, and socio-economic barriers. The authors emphasize the need for targeted public health interventions to enhance educational efforts about rabies prevention, improve vaccine access, and address economic barriers. While the manuscript provides valuable insights into rabies management and vaccination adherence in a specific Kenyan context, it could benefit from a more detailed exploration of study limitations and a stronger emphasis on actionable recommendations for public health strategies.

The study could benefit from broader contextualization by linking its findings to existing public health frameworks and rabies control programs in Kenya and comparing its results to previous interventions and their success rates to understand the correlation between awareness and vaccination uptake. Potential biases may arise in participant selection and self-reported vaccination behaviors, mainly due to social desirability bias common in survey studies. Additionally, the disconnect between high awareness and low vaccination adherence necessitates further exploration, as awareness alone does not ensure changes in health behaviors; interventions should address underlying beliefs, socio-economic barriers, and logistical challenges. Future research should focus on developing intervention strategies that engage the community and provide education tailored to demographic groups less likely to vaccinate their dogs, as literature indicates that targeted outreach can effectively bridge existing knowledge gaps.

I have a few more observations that the authors could consider. The line number should be inserted in the revised manuscript so that it is easy for the reviewer to make comments.

1. The introduction is lengthy, with repeats, and very generic. It must be shortened and better focused on the specific study. Consider following the logical flow of the scientific introduction. Stating the significance of addressing the specific gaps identified in previous studies would enhance the justification for the research.
2. The sample size is based on a well-defined statistical formula, which is commendable. The calculation and inclusion of a non-response rate are straightforward. However, details on how the structured questionnaires were validated or pretested should be included to ensure the reliability and validity of the data collection tools.
3. It is noted that structured questionnaires were employed alongside key informant interviews. However, a more detailed description of the questions, categories, or themes covered in the qualitative aspects would enhance transparency.
4. Data Analysis: The use of SPSS for data analysis is standard practice, although describing specific techniques (e.g., types of tests) applied would clarify the analytical approach. The qualitative data analysis process needs more elaboration. Details on coding, thematic analysis, or how themes were identified should be included.
5. Results: Avoid repeating data in the text already in the table except for the key findings.  The manuscript effectively presents results through tables, aiding in comprehension. Key statistics are included, although some sections could benefit from visual representation (e.g., graphs showing vaccination rates over time). The results are relevant and significant to the research question but could be organized more logically. Distinguishing between awareness and direct vaccination practice results could clarify the impact of awareness on behaviors.
6. The limitations of the study are not explicitly discussed.
7. Consider providing a more operationalized set of recommendations based on findings.
