# Supplementary material for: Factors influencing vaccination of dogs against rabies in South Mugirango Sub-County-Kisii County; Kenya
Source: PLOS Glob Public Health. 2025 May 9;5(5):e0004194. doi: 10.1371/journal.pgph.0004194 (PMC12063882; doi:10.1371/journal.pgph.0004194)
Supplement: Attachment — Submitted filename: Rebuttal statement.docx [file pgph.0004194.s003.docx]

Rebuttal statement

Abstract: No need to mention SPSS in the abstract.

Response:

Changes Made:

Removed “Data analysis was performed using SPSS version 26.0.”

Reworded the methodology section to reflect general statistical analysis techniques.

**Re-check keywords; use non-generic ones.**
**Response:**
**Changes Made:**

- Original keywords: Zoonotic diseases, Rabies, Vaccination.
- Revised keywords: Dog-mediated rabies, Zoonotic disease transmission, Rabies vaccination, Household awareness, Kisii County, South Mugirango, Public health interventions.

Reviewer 1:

The introduction is lengthy, with repeats, and very generic. It must be shortened and better focused on the specific study. Consider following the logical flow of the scientific introduction. Stating the significance of addressing the specific gaps identified in previous studies would enhance the justification for the research.

Response:

We agree with the reviewer’s comments regarding the length and repetition in the introduction. To address this, we have shortened the introduction and removed redundant points. We also focused more directly on the specific aim of our study, ensuring that the narrative better aligns with the logical flow of scientific writing. Furthermore, we have highlighted the gaps identified in previous studies and explained how our research seeks to address these gaps. This revision aims to improve the justification for the study and clarify its contribution to existing knowledge.

Changes Made:

The introduction has been revised to be more concise and focused.

Redundant content has been eliminated.

The rationale for the study has been strengthened by identifying gaps in existing research.

Reviewer 2:

Intro page 3, 2nd paragraph: need citation for “Many communities are unaware…”

Response:

We appreciate the reviewer’s suggestion to provide a citation for the statement “Many communities are unaware of the risks and prevention strategies associated with rabies.” We have now added the appropriate citation to this section, referencing relevant literature that supports the claim about community awareness (Ashwini et al., 2024; Mani & Madhusudana, 2013). This citation further strengthens the argument and aligns with the study’s objective of assessing awareness levels.

Changes Made:

A citation for the statement regarding community awareness has been added to the second paragraph of the introduction.

Intro page 3, paragraphs 4 and 5 do not add anything to the introduction narrative, especially where the aim is already spelled out in 3rd paragraph.

Response:

We agree with the reviewer’s observation that paragraphs 4 and 5 were somewhat redundant and did not contribute significantly to the introduction. These paragraphs have been revised and condensed. We have restructured the introduction so that the aim of the study is now introduced more directly in a clear and succinct manner without unnecessary repetition. The revised introduction improves the clarity and flow of the narrative.

Changes Made:

Paragraphs 4 and 5 have been revised and streamlined.

The study aim is now stated more clearly, without repetition.

Material and Method section

**Reviewer 2 Comments:**

1. **Citation for census numbers:** Census figures are now properly cited (KNBS, 2019; Kisii County Government, 2020).
2. **Why 400 households when sample size was 422?** Adjusted and clarified sample size to align with calculation and account for non-responses.
3. **Key Informant Selection:** Updated section to specify qualifications and inclusion/exclusion criteria for the KIIs.
4. **Equation Format:** Equations reformatted for clarity using a mathematical equation editor.
5. **22 Key Informants Justification:** Census approach explained to include all officers directly involved in rabies control.

**Reviewer 1 Comments:**

1. **Validation of Questionnaires:** Added pretesting details and expert validation for reliability and validity.
2. **Qualitative Themes and Questionnaire Description:** Expanded description of interview themes and socio-demographic factors.
3. **Statistical Techniques:** Elaborated analytical approaches for quantitative and qualitative data.

Results and discussion

Reviewer 2

**Reviewer’s Comment:**
The reviewer suggested the use of additional statistical tests or more comprehensive sensitivity analysis to verify the robustness of the findings.

**Response:**
. After further review, we have conducted additional sensitivity analyses, including using alternative statistical methods such as logistic regression to check the robustness of the relationships between socio-demographic factors and vaccination adherence. I have included these results in the revised manuscript, with additional discussion on how these alternative tests corroborate the original findings. These revisions are now reflected in the Methods and Results sections.

**Reviewer’s Comment:**
The reviewer pointed out that the interpretation of the results, particularly for non-significant findings, could be expanded.

**Response:**
we agree that a more nuanced interpretation of non-significant results is necessary. I have revised the discussion section to provide a more thorough exploration of the non-significant associations, specifically explaining that the absence of a statistically significant relationship does not negate the relevance of these factors in the context of vaccination adherence. This revision aims to ensure that readers understand the implications of both significant and non-significant findings in the broader context of public health interventions.

**Reviewer’s Comment:**
The reviewer indicated that while the tables are well-organized, some columns are not clearly labeled, and the statistical results could benefit from more explanation.

**Response:**
we have revised the tables to ensure that each column is clearly labeled and the statistical results are presented with more detailed explanations. Footnotes have been added to clarify the significance of the tests and the definitions of terms used in the tables. I hope these revisions make the presentation of data clearer for readers and improve the manuscript’s overall accessibility.

**Reviewer’s Comment:**
The reviewer recommended a more thorough discussion of the study's limitations, particularly related to sampling bias and the potential for self-reported data bias.

**Response:**
 In the revised manuscript, we have expanded the limitations section to include a more detailed discussion of potential biases in the study, specifically addressing the sampling method and the reliance on self-reported data. I

**Reviewer’s Comment:**
The reviewer asked for a more detailed discussion on how the findings can inform vaccination campaigns and public health policy.

**Response:**
we have significantly expanded the conclusion to focus on how the findings can inform practical interventions, particularly by targeting specific socio-demographic groups that may be under-represented in vaccination coverage. This includes discussing strategies for improving access to vaccination services in rural areas or addressing knowledge gaps about rabies vaccination in specific demographic groups. These revisions emphasize the study’s public health relevance and potential impact.

**Reviewer’s Comment:**
The reviewer suggested including more recent studies related to rabies vaccination adherence.

**Response:**
we have reviewed the reference list and incorporated several recent studies that address rabies vaccination adherence, particularly those conducted in similar settings or populations.

Reviewer 2

"The reviewer noted that the chi-square test results did not show significant associations for some socio-demographic factors, particularly gender, marital status, and the number of children."

the lack of significant associations for some factors, such as gender and marital status, is an important finding in itself. We have revised the discussion section to emphasize that non-significant results are valuable and should be interpreted as such.

: "There seems to be insufficient discussion about the potential implications of non-significant results."

We have expanded the discussion to address the implications of non-significant results, particularly in the context of how socio-demographic factors interact with vaccination adherence. I also clarified that non-significant findings in this study suggest that other contextual factors, beyond those considered here, may play a more substantial role in influencing adherence.

"The tables are well-organized, but the presentation of the statistical results could benefit from clearer labeling and explanations."

Response: I have revised the tables to include clearer labels for the statistical tests used, and I have added footnotes to explain the key terms and statistical results more thoroughly.

"The study could benefit from a more detailed discussion on the limitations of the study design, particularly regarding the sampling method."

added a section in the limitations discussion where I address the sampling method. I agree that a more nuanced consideration of potential biases related to the study design, such as the non-random sampling approach and potential regional differences, is important.

"There is a concern about the potential biases in self-reported data, particularly in the assessment of vaccination adherence."

I have acknowledged the potential for self-report bias in the data collection process. In the revised manuscript, I have elaborated on how this bias could have impacted the results and discussed ways in which future studies could mitigate this issue, such as through triangulation with official vaccination records or more objective measures of vaccination status.

"The study provides valuable insights into the socio-demographic factors influencing rabies vaccination adherence, but I would like to see more discussion on how these findings can be used to design interventions."

. I have highlighted specific socio-demographic factors, such as educational level, occupation, and income, which could be targeted in intervention strategies aimed at improving vaccination adherence in the community.

"The reference list is generally well-rounded, but I recommend including more recent studies on rabies vaccination."

I have reviewed the reference list and added several more recent studies related to rabies vaccination adherence. This helps to ensure the study is grounded in the most current literature on the subject.
