# Supplementary material for: Factors influencing vaccination of dogs against rabies in South Mugirango Sub-County-Kisii County; Kenya
Source: PLOS Glob Public Health. 2025 May 9;5(5):e0004194. doi: 10.1371/journal.pgph.0004194 (PMC12063882; doi:10.1371/journal.pgph.0004194)
Supplement: Attachment — Submitted filename: Rebuttal Letter.docx [file pgph.0004194.s004.docx]

**Rebuttal Letter for Manuscript Entitled Factors Influencing Vaccination of Dogs against Rabies in South Mugirango Sub-County, Kisii County, Kenya- PGPH-D-24-02047R1**

Dear Editor,

We sincerely appreciate the valuable feedback from the reviewers. Below, we provide a point-by-point response to each of the comments and outline the revisions made.

Reviewer 1

Comment 1: The abstract currently presents the study's content, but it could benefit from a more explicit emphasis on the central aim of the research. Since the study's primary objective is to assess factors influencing dog vaccination against rabies among household heads, I suggest highlighting this key focus more prominently.

Response: We have revised the abstract to emphasize the study's central aim more explicitly. The first sentence now clearly states the research focus on assessing factors influencing dog vaccination among household heads in South Mugirango. Additionally, we have refined the language to align with academic standards for improved clarity.

Reviewer 2

General Comments

Comment 1: Check for grammar and syntactical errors like missing commas, and periods. There are multiple missing spaces between sentences. The manuscript should have a consistent writing style and formatting. Please check with a language editor if necessary.

Response: We carefully reviewed and revised the manuscript for grammar, punctuation, and formatting consistency. A professional language editor was consulted to ensure academic clarity and coherence.

Comment 2: No data availability statement was found in the manuscript.

Response: A data availability statement has now been included in the manuscript as per the journal's guidelines.

**Introduction**

Comment: The last three paragraphs describe the impacts and aims of the study. Try to combine or shorten them.

Response: We have condensed the last three paragraphs of the introduction by integrating the impact discussion into the study rationale. This ensures a more concise and structured introduction.

Materials and Methods

Comment 1: “Participants for the quantitative survey were recruited based on the inclusion criteria of being household heads who owned at least one dog and provided informed consent.” This is repeated twice—once in recruitment of participants and once in inclusion criteria. Remove one.

Response: We have removed the redundant sentence while ensuring clarity on participant selection.

Comment 2: “Statistical significance was set at p<0.05p < 0.05p<0.05.” Repeated terms?

Response: This was a typographical error, and we have corrected it.

Comment 3: “Qualitative data were analyzed thematically, with data grouped into emerging themes.” Explain how? Given the reference to a previous study, it would be better to summarize it in a sentence or two.

Response: We have expanded on the thematic analysis process by specifying the framework used (Braun & Clarke, 2006) and describing how themes were generated and validated.

Comment 4: “In South Mugirango Sub-County, several socio-demographic factors play a role in shaping the immunization practices of Household Heads (HHs) concerning their dogs.” Is this the author’s expertise?

Response: We have clarified that this observation is based on study findings and existing literature rather than personal expertise.

Results

Comment 1: Paragraphs between: “For gender, the data revealed…. Respondents with no formal education had negligible representation, with 0% adhering.” These are redundant. Instead, add interpretation of the findings in Table 1.

Response: The redundant text has been removed, and we have included a concise interpretation of Table 1 findings.

Comment 2: “The logistic regression analysis aimed to identify socio-demographic factors that influence adherence to dog vaccination in South Mugirango.” How? What is the dependent variable? What do the coefficients mean? Are they odds ratios for vaccination? Please explain the use and application of logistic regression to help readers understand this analysis.

Response: We have revised this section to clearly define the dependent variable, explain the logistic regression model, and clarify that the coefficients represent odds ratios for vaccination adherence.

Comment 3: Table 2: Are the coefficients and CI on a log scale or natural scale? Seems to me that the coefficients have not been exponentiated while the CI is exponentiated. Why? No mention in footnotes.

Response: We have added clarification in the footnotes, specifying that the coefficients are on a log scale while the confidence intervals are exponentiated.

Comment 4: “The interviews with livestock and public health officers corroborated the quantitative findings, emphasizing the significant role of socio-demographic factors in vaccination adherence.” Where are the specific results of the interview pertaining to this?

Response: We have added direct references to qualitative findings from key informant interviews to support this statement.

Discussion

Comment: A lot of information in the discussion, especially pertaining to the indicators of socio-demographic characteristics, needs to be mentioned in the Introduction section at least. Refer to my previous comment: “Is this author’s expertise?” The information in the discussion addresses this comment but comes too late in the text. Major restructuring of the introduction with respect to the discussion section is necessary to make both sections coherent.

Response: We have moved relevant socio-demographic background information from the discussion to the introduction for better coherence and logical flow.
