# Supplementary material for: Factors influencing vaccination of dogs against rabies in South Mugirango Sub-County-Kisii County; Kenya
Source: PLOS Glob Public Health. 2025 May 9;5(5):e0004194. doi: 10.1371/journal.pgph.0004194 (PMC12063882; doi:10.1371/journal.pgph.0004194)
Supplement: Attachment — Submitted filename: Response_Letter_to_editor.docx [file pgph.0004194.s005.docx]

**Rebuttal Letter for Manuscript Entitled Factors Influencing Vaccination of Dogs against Rabies in South Mugirango Sub-County, Kisii County, Kenya- PGPH-D-24-02047R1**

Dear Editor,

We appreciate the time and effort in evaluating our manuscript. Below, we provide a detailed response to each comment and outline the changes made in our revised manuscript.

**Reviewer Comments and Responses:**

**Abstract: You included more text in the introduction section and less text in the results section. Suggest including more information in the results section and reducing words in the introduction section.**
**Response:** We have revised the abstract to balance the sections by condensing the introduction and expanding the results. Additional statistical results and key findings have been incorporated to provide a clearer summary of our study’s outcomes.

**Results: Suggest including the study period.**
**Response:** The study period (January 2019 to January 2020) has now been explicitly mentioned in the study design section section for clarity.

**Results: How many key informant interviews were conducted? Mention this explicitly.**
**Response:** We have clarified that a total of 22 key informant interviews (KIIs) were conducted in the study. This information is now highlighted in both the methods and results sections.

**Results: I did not find the revised Table 1. It is deleted. Why?**
**Response:** Table 1 was renamed to table 2 in the result section on vaccination adherence.

**Every study has limitations. Please add a paragraph for limitations after the discussion section.**
**Response:** A new limitations section has been added after the discussion. This section acknowledges recall bias, potential social desirability bias in self-reported data, and logistical constraints that may have affected participant selection and generalizability.

**Conclusions look too long. The first paragraph is fine. Suggest relocating the last three paragraphs to the discussion section.**
**Response:** We have shortened the conclusion by retaining only the first paragraph while integrating the last three paragraphs into the discussion section where they are more appropriate.
